# Parallel evolution of character displacement driven by competitive selection in terrestrial salamanders

**DOI:** 10.1186/1471-2148-10-72

**Published:** 2010-03-10

**Authors:** Dean C Adams

**Affiliations:** 1 Department of Ecology, Evolution, and Organismal Biology and Department of Statistics, Iowa State University, Ames IA, 50011, USA

## Abstract

**Background:**

Parallel evolution can occur when common environmental factors exert similar selective forces on morphological variation in populations in different geographic localities. Competition can also generate morphological shifts, and if competing species co-occur in multiple geographic regions, then repeated instances of competitively-driven morphological divergence (character displacement) can occur. Despite the importance of character displacement for inferring the role of selection in morphological evolution however, replicated instances of sympatric morphological divergence are understudied.

**Results:**

I tested the hypothesis that interspecific competition generated patterns of parallel morphological divergence in multiple geographic locations where two competing salamander species, *Plethodon jordani *and *P. teyahalee*, come into contact. I used geometric morphometrics to characterize head shape and found ecological character displacement in sympatric localities on each of three distinct mountains (geographic transects), where sympatric specimens displayed greater cranial differences and an increase in cranial robustness as compared to allopatric specimens. Using a recently developed analytical procedure, I also found that the observed morphological evolution within each species was consistent among transects; both in the total amount of morphological change as well as the direction of evolution in the morphological data space. This provided strong statistical evidence of parallel morphological evolution within species across replicate geographic transects.

**Conclusions:**

The results presented here reveal that the morphological evolution of each species followed a common evolutionary path in each transect. Because dispersal between sympatric locations among transects is unlikely, these findings suggest that the repeated instances of character displacement have evolved in situ. They also suggest that selection from competitive interactions plays an important role in initiating sympatric morphological divergence in these species, and drives parallel sympatric morphological divergence between species.

## Background

A major goal in evolutionary biology is to understand how disparate taxa respond to similar selection pressures. In some instances, distinct evolutionary responses are observed in taxa experiencing common selective environments [1], implying that the unique histories of organisms can play a large role in shaping the path of evolution [2]. Other times, common selective pressures elicit similar (parallel) evolutionary responses, suggesting that the evolutionary process can be repeatable. Some of the more tantalizing examples of parallel evolution found in vertebrates include the evolution of distinct body forms of freshwater fishes in different habitats [3-6], lizard ecomorphs on different islands [7-9], recurring phenotypes of cichlids in African Rift Lakes [10-12], and distinct body forms and life history traits found along predation gradients [13-16]. Such examples reveal natural selection's strong role in shaping trait evolution [17,18], and suggest that repeated patterns of parallel evolutionary change may contribute to diversification at higher scales [19,20].

Parallel evolution can occur when common environmental factors (e.g., salinity: [21,22]) or other habitat-specific attributes (e.g., differing predation levels: [16]) exert similar selective forces on morphological and life history variation in populations in different geographic localities. In addition, because evolutionary changes are affected by underlying patterns of genetic variation [23-25], parallel evolution can also occur if, within populations, the genetic covariance patterns among traits are similar. Under such a scenario, the common genetic architecture exhibited within populations could give rise to similar patterns of phenotypic evolution among populations, resulting in parallel morphological evolution. When this is the case, trajectories of phenotypic evolution may also be expected to align closely with the major direction of genetic variation, or g_max_, [26-28].

Another factor that plays a key role in promoting morphological change is interspecific competition, which frequently enhances the morphological differences between competitors (character displacement: [18,29-32]). Competition can also generate patterns of parallel evolution, if the two competing species co-occur in multiple geographic regions, and if the competition between them results in repeated instances of competitively-driven morphological divergence in each region. Indeed, replicated patterns of morphological divergence due to competition have been identified in a number of systems [4,6,33,34]. These examples demonstrate the consistency of competitively-based selection pressures in nature, and provide strong evidence of competition's role in generating parallel evolutionary diversification across communities.

The salamanders of the genus *Plethodon *represent an attractive study system for identifying replicate patterns of morphological evolution resulting from interspecific competition. *Plethodon *are widely distributed in the forests of North America [35]. Extensive field collecting at thousands of localities has rigorously documented their geographic distributions, and their species-level phylogeny is becoming well-resolved [36,37]. Further, decades of ecological research has shown that interspecific competition is prevalent in *Plethodon *[38-41], and likely influences community structure at both a local and regional scale [42]. In addition, morphological changes are often associated with competition in many communities [29,43-46], suggesting that morphological evolution can be driven by competitive interactions in this group.

**Figure 1:**
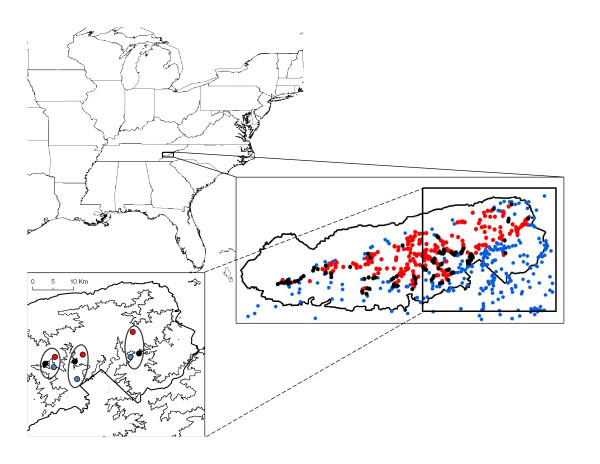
**Map of study region**. Upper Left: Map of the eastern United States. Right Center: Map of the Great Smoky Mountain National Park, with collection localities from the USNM collections shown (red: *P. jordani *allopatric localities; blue: *P. teyahalee *allopatric localities; black: sympatric localities for both species). Lower Left: Map of the study region, with the locations of three geographic transects circled: left = Taywa Creek localities; center: Kephart Prong localities; right:, Heintooga Ridge localities. Allopatric and sympatric localities are designated with the same colors as in the central figure. The boundary of the Great Smoky Mountain National Park is shown, and the light line denotes 1,200 m in elevation.

In the southern Appalachian Mountains, the interactions of two species, *Plethodon jordani *and *P. teyahalee*, have been particularly well characterized. These species exhibit altitudinal distributions, with *P. jordani *inhabiting higher elevations and *P. teyahalee *inhabiting lower elevations. Although both species overlap widely in the western portions of their ranges, in the eastern Great Smoky Mountains, their overlap is more limited, occurring in a narrow zone at mid-elevations (Figure 1). In this region, most localities that have been sampled are allopatric, containing one or the other species, and the sympatric zone is not continuous, but is restricted to particular mountain ridges [47,48]; a pattern suggestive of allopatric individuals colonizing sympatric localities. Considerable ecological and behavioral work has been performed on these sympatric, and neighboring allopatric populations, and has demonstrated that interspecific competition is a dominant force in communities where both species are found sympatric with one another [39,47,49-52].

Recently, a morphological analysis revealed ecological character displacement in head shape between the two species [44]. In that study, greater head shape divergence was identified between sympatric populations of these species at several locations in the eastern Great Smoky Mountains, relative to what was observed between neighboring allopatric populations. The localities examined originated from three distinct mountain transects where prior behavioral work had identified competitive interactions between these species [39,49,51,52]. However, while sympatric morphological differences were identified, it is unclear whether the trajectories of evolutionary change exhibited by each species from allopatry to sympatry were concordant among geographic transects. Therefore it is not known if competitive interactions between species have resulted in repeated instances of parallel morphological evolution in this system. The purpose of this study is to examine patterns of character displacement between *P. jordani *and *P. teyahalee *across multiple geographic transects to determine whether in fact they are concordant. Specifically, I test the hypothesis that interspecific competition has driven repeated patterns of morphological change in each species across distinct regions, resulting in parallel evolution of character displacement in regions of co-occurrence.

## Results

**Figure 2:**
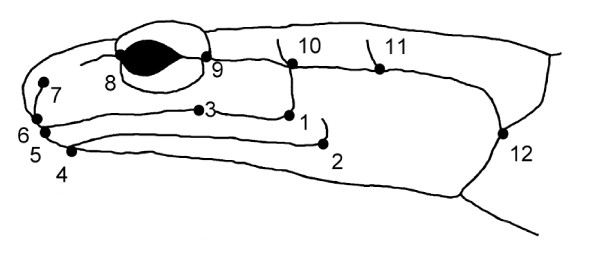
**Landmarks used to characterize head shape of salamanders**. Positions of 12 anatomical landmarks used to quantify head shape in *Plethodon *(image from [44]).

I quantified head shape of specimens from three distinct mountain transects in the Great Smoky Mountains (Figure 1). Each transect contained an allopatric locality for *P. jordani *and an allopatric locality for *P. teyahalee *(at high and low elevations respectively), and one sympatric locality at mid-elevations where both species co-occurred. Head shape was characterized with a set of 12 landmarks (Figure 2) and geometric morphometric methods [53-55]. A multivariate analysis of variance (MANOVA) on the resulting shape variables revealed significant morphological differences between species and between locality types, and identified a significant species × locality interaction (Table 1). Previous analyses [44] found morphological differences were greater in sympatric localities than in allopatric localities, a pattern consistent with character displacement. The results presented here were concordant with this result, but also provided an assessment of patterns of divergence among geographic transects.

**Table 1:** Results from multivariate analysis of variance (MANOVA) of head shape across three geographic transects of *Plethodon jordani *and *P. teyahalee *in the eastern Great Smoky Mountains.

Factor	**Df**_**Factor**_	Pillai's Trace	Approx. F	Df_**num**_, Df_**denom**_	*P*
Species	1	0.741	48.874	18, 307	< 0.0001
Locality Type	1	0.794	65.612	18, 307	< 0.0001
Geographic Transect	2	0.783	11.015	36, 616	< 0.0001
Species × Locality	1	0.519	18.373	18, 307	< 0.0001
Species × Transect	2	0.289	2.888	36, 616	< 0.0001
Locality × Transect	2	0.338	3.482	36, 616	< 0.0001
Species × Locality × Transect	2	0.161	1.499	36, 616	0.0327

To compare patterns across geographic transects, I used a recently developed analytical approach to quantify and compare vectors of morphological evolution [56-58]. When morphological patterns were examined among transects with this procedure, no differences were found in the magnitude of morphological evolution exhibited across transects for either *P. jordani *or for *P. teyahalee *(Table 2). This implied that the amount of morphological evolution within species was consistent across the geographic transects. Similarly, there were no differences in the orientation of morphological evolution across transects for either species (Table 2), implying that morphological evolution within species proceeded in a similar direction across transects. Summary variance statistics [57] were in accord with the pairwise analyses, and were not significant for either the magnitude of morphological evolution or their direction of evolution in the morphological data space (Var_size _= 0.0000625, P = 0.1957; Var_orient _= 59.4819, P = 0.5058). Finally, the observed patterns were not the result of sexual dimorphism: significant differences in head shape were identified between the sexes (Pillai's trace = 0.503, F = 5.91, P < 0.0001), but when sex was included in the model, patterns of morphological evolution were consistent with what was reported above (results not shown). Thus, sexual shape dimorphism did not bias estimates of morphological evolution from allopatry to sympatry.

**Table 2:** Statistical comparisons of evolutionary vectors of morphological change.

	Vector Magnitude			Vector Orientation		
**A: *P. jordani***	**HR**	**KP**	**TC**	**HR**	**KP**	**TC**

HR		0.1849 NS	0.3192 NS		0.6074 NS	0.3665 NS
KP	0.01689		0.0309 NS	26.785		0.4071 NS
TC	0.00871	0.02560		31.502	41.545	

						

**B: *P. teyahalee***	**HR**	**KP**	**TC**	**HR**	**KP**	**TC**

HR		0.3363 NS	0.8106 NS		0.7965 NS	0.5579 NS
KP	0.00871		0.3261 NS	19.506		0.5069 NS
TC	0.00253	0.01224		25.033	34.136	

Upper block: results for *Plethodon jordani*: Lower block: results for *P. teyahalee*. Pairwise differences in vector magnitude and vector orientation are shown below the diagonal, and their significance levels (based on 9,999 random permutations) are shown above the diagonal. Geographic transects are designated as: HR = Heintooga Ridge, TC = Taywa Creek, KP = Kephart Prong. Significance levels are compared to a Bonferroni corrected error rate of α = 0.0167. No pairwise comparison was significant.

**Figure 3:**
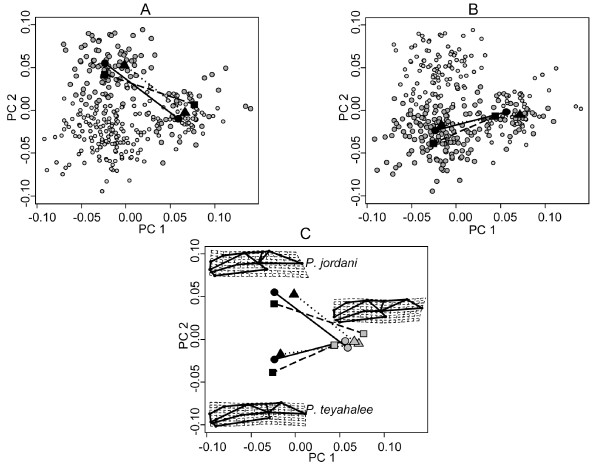
**Principal components plot of head shape variation**. (A) Data for *Plethodon jordani *are emphasized, and (B) data for *P. teyahalee *are emphasized. The first two principal component axes explain 48.6% of the total variation in head shape. For each panel, individuals of the focal species are shown in large, gray symbols, the other species in light symbols. Also displayed are least-squares means for each population, and vectors of phenotypic evolution for each geographic transect. Geographic transects are shown as: Heintooga Ridge (solid lines and circles), Taywa Creek (dashed lines and squares), Kephart Prong (dotted lines and triangles). (C) Phenotypic evolution vectors for each transect for both species in this study. Phenotypic means for allopatric populations are shown in gray; phenotypic means for sympatric localities are shown in black. Thin-plate spline deformation grids depict typical head shapes for sympatric populations of *P. jordani *and *P. teyahalee*. A single deformation grid represents the allopatric populations for both species.

When viewed in the principal components plot, the evolutionary patterns of morphological change were evident. I found that all three evolutionary vectors for *P. jordani *were oriented similarly in the morphological data space, indicating that the direction of morphological evolution from allopatry to sympatry was concordant. These vectors were also of similar length, revealing that the amount of morphological evolution exhibited from allopatry to sympatry was consistent across transects as well (Figure 3A). Likewise, the three evolutionary vectors for *P. teyahalee *were oriented similarly, and identified a concordant amount of morphological evolution from allopatry to sympatry in each transect (Figure 3B). However, the evolutionary vectors for *P. teyahalee *were oriented differently from those for *P. jordani*, indicating that the direction of morphological evolution differed between species (the angular difference between species vectors was 47.71°; P_rand _= 0.0001). Specifically, the allopatric endpoints of all evolutionary vectors were in a similar location in shape space (indicating similar morphologies in each transect), while the sympatric endpoints of all evolutionary trajectories were found in different locations in shape space for each species, signifying head shape differences between species in sympatry. As in previous analyses [44], the morphological evolution from allopatry to sympatry in both species described a general increase in cranial robustness, with sympatric specimens displaying relatively more elongate jaws, and exhibiting a relative expansion in the posterior region of the head (Figure 3C). In addition, the jaw was relatively thicker in sympatric *P. jordani *as compared to sympatric *P. teyahalee *(Figure 3C).

## Discussion

The repeated evolution of similar morphological traits in species inhabiting common environments has long been treated as evidence of adaptation [17,18,59], and reveals that environmentally-induced selection pressures can generate parallel patterns of diversification. Similarly, interspecific competition can drive parallel evolution, when the competitive mechanisms between species are comparable in distinct locations where competing species co-occur. In this study, I examined morphological divergence between two competing salamander species, *Plethodon jordani *and *P. teyahalee*, in several distinct regions in the eastern Great Smoky Mountains to determine whether trajectories of evolutionary change within species were concordant among geographic transects. I found significant morphological evolution in head shape within species from allopatry to sympatry, revealing an evolutionary response to interspecific competition. I also found that morphological differences were enhanced in sympatry, a pattern consistent with character displacement. Within species, the morphological evolution exhibited was concordant among distinct geographic transects, identifying a common pattern of morphological change that implied parallel evolution. Finally, considerable prior work on this system has documented interspecific competition at these localities [39,49,51], has ruled out alternative explanations for the observed patterns [44], and has provided evidence of a link between morphological variation and aggressive behavior in this system [44]. Thus, for this system there is empirical support for five of the six criteria for character displacement (i & ii: chance and alternative explanations ruled out, iii: independent evidence of competition, iv: association of morphology to putative selective force, vi: comparable relevant ecological parameters across localities [6,60]). Together these suggest that the morphological divergence observed in sympatry represents replicated instances of character displacement, and that selection from competitive interactions has driven parallel evolution of morphological divergence in these species.

In addition to parallel evolution, shared evolutionary history can also produce common morphological patterns across localities [8,61]. This arises when morphologically similar species found in distinct locations are also phylogenetically closely related. For the present study, this scenario would occur if the sympatric populations were more closely related to one another (within species) than they were to neighboring allopatric localities, suggesting dispersal between sympatric locations as the cause of the similar morphological patterns. While this remains a possibility, a number of factors make it unlikely. First, as discussed previously, *Plethodon *are highly philopatric; they have small home ranges, exhibit strong homing behavior [62], and genetic differentiation among geographically proximate *Plethodon *populations, even at distances as small as 200 meters, has been identified [63]. Second, the straight-line distances between sympatric localities in this study are between 25 and 80 times larger than the maximal known dispersal distance for *Plethodon *[64], and the geographic distances between sympatric localities at a constant elevation are considerably larger. Finally, despite extensive sampling of the geographic ranges of these two species (1,097 total localities and 448 localities in the Great Smoky Mountain National Park alone: USNM collections: Figure 1), no additional sympatric regions have been identified between the three transects studied here. All other known localities between transects are allopatric, and contain only one or the other of these species (the Taywa Creek sympatric zone does extend a few kilometers beyond the study location, but this represents less than one quarter of the approximately 26 kilometers between this transect and Kephart Prong when traversed at mid-elevations). Thus, any dispersal from sympatric localities would have to proceed through intervening regions of allopatry. Taken together, this strongly suggests that the sympatric localities studied here originated from dispersal from neighboring allopatric populations of each species, and not from other sympatric populations. As such, these localities can be treated as independent origins of sympatry, and parallel evolution is the most likely explanation for the observed morphological patterns.

If it is true that these patterns represent independent origins of parallel sympatric diversification, how can they be explained in terms of the evolutionary processes that may have caused them? Several evolutionary explanations are possible, which may act separately, or in concert to produce such patterns. One possibility is that common genetic covariance among traits has directed the path of evolution in common directions among transects. This hypothesis suggests that genetic covariance serves as a constraining force on the effect of selection [65], resulting in correlated patterns between genetic covariation and the direction of morphological change. Direct tests of this hypothesis require an estimate of the genetic covariance matrix within populations, which unfortunately are not available for this system, as large scale quantitative genetics experiments have not been performed on these long-lived species. An examination of the phenotypic covariance matrix as a surrogate for the genetic covariance matrix did show that the observed vectors of evolutionary change from allopatry to sympatry were not always aligned with the main direction of phenotypic variation within populations (results not shown), though the extent to which genetic and phenotypic covariation are correlated in these species has yet to be determined. Future studies should examine the degree to which patterns of genetic covariation affect the direction of phenotypic evolution in these species.

Another possible explanation is that the observed patterns of parallel diversification are an evolutionary response to selection. Under this scenario, similar selection pressures occur in distinct geographic localities, resulting in common evolutionary responses across transects (note that both selection and genetic covariance often interact to affect diversification patterns). For the two species examined here, the common selective pressures are likely the result of interspecific competition. This hypothesis is based on the observation that interspecific competition and aggressive behavior is known to be intense between these two species in these sympatric localities [39,49-52]. Further, there is a significant correlation between aggressive behavior and head shape in these locations [44], suggesting a possible causal link between levels of aggression and changes in cranial morphology in sympatric localities. The latter observation is critical; as it provides a putative functional link between the cause of selection and the evolutionary response exhibited in morphological change.

Taken together, my findings suggest that selection resulting from competitive interactions plays an important role in initiating sympatric morphological divergence, and appears to be the driving force responsible for parallel evolution of character displacement across transects. Given the important role of interspecific competition for understanding morphological evolution in this system, what does this suggest about broader patterns of morphological evolution in the genus *Plethodon*? Based on the prevalence of competition among species in the genus *Plethodon *[38,41], I hypothesize that parallel evolution of character displacement may be more widespread. First, there are many species pairs that exhibit similar geographic patterns to those seen in *P. jordani *and *P. teyahalee*, with geographically distinct sympatric localities found throughout their contact zone. This is particularly the case for species in the *P. cinereus *species group, where the wide-ranging *P. cinereus *is found sympatric in multiple locations with other competing species (e.g., *P. nettingi*: [66,67]; *P. hubrichti*: [68]; *P. hoffmani*: [29,69]). Further, morphological divergence has been documented in certain sympatric localities for some of these systems [29,45,46,69]; but see [70]. Thus, if interspecific competitive mechanisms are relatively consistent across sympatric localities, it is possible that morphological evolution could proceed similarly in multiple locations for one or both competing species. This hypothesis should be examined in future studies.

Finally, examining these patterns in light of phylogenetic history and other macroevolutionary trends may lead to important insights into the relationship between competition, morphology, ecology, and speciation in *Plethodon*. As noted previously, interspecific competition is prevalent in *Plethodon *[38,40,41], and morphological evolution is often associated with interspecific interactions [29,45]. Likewise, phylogenetic niche conservatism appears common [71,72], as sister species tend to exhibit similar environmental tolerances. Further, this species-specific signal in niche use has been posited as having a positive influence on allopatric speciation in *Plethodon*, as closely related species exhibit similar environmental tolerances but frequently display distinct geographic ranges [71]. The morphological patterns described here also exhibit a strong species-specific signal, and extending these analyses to a broader array of *Plethodon *species would establish whether or not closely related species exhibit similar morphological responses to interspecific competition. If such patterns were identified, they would suggest that interspecific competition, and subsequent morphological diversification, may play an important role in the proliferation of the clade. Under these circumstances, comparing patterns of morphological evolution to patterns of niche conservatism would allow one to determine the relative contributions of competitively-induced morphological change and environmental adaption to speciation and diversification in the group.

## Conclusions

This study characterized patterns of morphological evolution from allopatry to sympatry in three mountain transects where two salamander species, *Plethodon jordani *and *P. teyahalee*, come into contact. I showed that in each transect, sympatric morphological divergence (character displacement) had likely evolved as a result of interspecific competition. I further showed that within species, the magnitude and direction of morphological evolution was consistent, and that alternative explanations cannot fully explain the observed patterns. The findings presented here reveal that parallel evolution of character displacement has occurred in these species, and suggest a strong role for selection on the evolution of diversification in this group.

## Methods

A total of 336 adult salamander specimens from the United States National Museum (USNM) were used in this study. These were part of a previous analysis [44], and were collected from several localities in three altitudinal transects in the eastern Great Smoky Mountains (Figure 1). Each transect was found on a distinct mountain ridge: Heintooga Ridge (HR) on Balsam Mountain, Kephart Prong (KP) on Richland Mountain, and Taywa Creek (TC) on Hughes Ridge (for additional locality information see [39,51,52]). In each transect, localities where both species were present (i.e., sympatric localities) were found at mid-elevations (approximately 1200 - 1500 m), allopatric localities of *P. teyahalee *at low elevations below the sympatric localities, and allopatric localities of *P. jordani *at high elevations above the sympatric localities. Thus, each transect represented a competitive gradient between allopatric localities of one species and sympatric localities containing both species. Sympatric localities of these transects were separated by straight-line distances of 7.8 km (KP-TC), 16.4 km (HR-KP), and 24.2 km (HR-TC) respectively (Figure 1). No additional sympatric localities between these transects have been found, and because *Plethodon *have low dispersal abilities [63], with 300 m being the maximal dispersal distance ever recorded [64], dispersal between sympatric localities is improbable. Rather, it is more likely that sympatric localities were derived from neighboring allopatric populations within transects.

To quantify morphology, I used geometric morphometric methods [53-55]. These methods quantify the shape of anatomical objects from the coordinates of repeatable locations, after the effects of non-shape variation are mathematically held constant. First, the locations of 12 anatomical landmarks were recorded from the head and jaw of each specimen (Figure 2). Specimens were then optimally aligned using a generalized Procrustes superimposition [73], and shape variables were generated using the thin-plate spline [74] and standard uniform components [75]. For the present analysis, variation in the gape of the jaw was also taken into account. This was accomplished using the separate subset method, where shape variables for the skull and jaw were generated separately, and were subsequently combined to provide an overall description of head shape (see [44,76]). A total of 18 shape variables represented the head shape of each specimen, and were used in all multivariate analyses. The sex of each specimen was also determined through gonadal inspection. Sex was reliably determined for 328 individuals (176 males and 152 females).

I performed a number of statistical tests to examine patterns of morphological evolution within and among transects. First, to compare head shape variation among species, localities, and transects, I conducted a full factorial multivariate analysis of variance (MANOVA), with species, locality type (allopatry vs. sympatry), and transect as main effects. I then tested the hypothesis of parallel evolution by statistically comparing patterns of morphological evolution from allopatry to sympatry within species among transects. To accomplish this, I first quantified the observed morphological evolution for each species in each transect as a multivariate vector, defined as the difference between allopatric and sympatric least-squares means from the MANOVA. Next, I calculated the magnitude of each evolutionary vector and its orientation in the morphological data space [56,58], and pairwise differences in these values were obtained. The within-species pairwise differences in magnitude and orientation (three per species) were then statistically evaluated using a residual randomization procedure with 9,999 iterations as follows. Briefly, a reduced model lacking the species × locality × transect interaction term was calculated, from which predicted values (i.e. least squares means) and residual values were obtained. The residuals were then randomized, and added to predicted values. The full model was recalculated with these data, random evolutionary vectors were estimated, and pairwise differences in their magnitude and orientation were calculated. The observed within-species pairwise differences were then compared to distributions of random values to assess their significance (for full statistical details see [57,58]). Statistical assessments were made at an experiment-wise error rate of α = 0.05 using Bonferroni correction. For this study, residual randomization was used rather than other resampling procedures because it has superior statistical power for assessing factorial designs [77]. Finally, because sexual size dimorphism is present in many plethodontids [78], the above analyses were repeated with sex included as a term in the model, to account for possible sexual dimorphism in head shape. Small sample sizes of each sex in some localities precluded separate analyses of males and females (<10 in each sex for most allopatric localities).

The analyses above provided a pairwise assessment of evolutionary concordance among transects. However, if the overall variance among evolutionary vectors was also small, this would reveal broader evidence of parallel evolutionary change across the set of transects. To assess this quantitatively, I calculated summary variance statistics from the set of within-species pairwise differences in vector magnitudes and the set of within-species pairwise differences in vector orientation [57], which were then statistically assessed using the residual randomization procedure described above. Patterns of morphological evolution were also examined using scores along the first two principal components (PC) using the shape variables as data, and thin-plate spline deformation grids [74] were used to facilitate biological interpretation of head shape changes. All statistical analyses were performed in R 2.81 [79].
